# Delayed neurotoxicity in HER2-positive breast cancer: a case series on combined SRS and T-DM1 treatment

**DOI:** 10.3389/fonc.2024.1448593

**Published:** 2024-10-01

**Authors:** Menekse Turna, Hale Başak Çağlar

**Affiliations:** Department of Radiation Oncology, Anadolu Medical Center, Gebze, Kocaeli, Türkiye

**Keywords:** HER-2 breast cancer, stereotactic radiotherapy, brain metastasis, trastuzumab emtansine, radionecrosis

## Abstract

This case series presents four instances of late neurotoxicity observed in HER2-positive breast cancer patients with brain metastases following treatment with stereotactic radiosurgery (SRS) and subsequent trastuzumab emtansine (T-DM1) therapy. Despite initial control of intracranial disease, patients experienced neurological deterioration months to years post-treatment. Radiological assessments revealed distinct patterns consistent with radiation necrosis, particularly in areas previously treated with SRS and subsequent T-DM1 administration. These changes, characterized by enlarging cystic masses with hemorrhagic components, emphasize the importance of vigilant monitoring in patients undergoing combined SRS and T-DM1 therapy for brain metastatic breast cancer. This report underscores the need for further investigation into the long-term effects of combining SRS with novel systemic therapies, particularly in HER2-positive breast cancer patients with brain metastases. Understanding and mitigating late neurotoxicity are critical for optimizing treatment strategies and improving patient outcomes.

## Introduction

Breast cancer is one of the leading cancer types that can develop brain metastasis, and the risk of brain metastasis (BM) in Her-2-positive and triple-negative breast cancer subtypes is significantly higher, with a lifetime risk of 40-50% compared to other subtypes (1–3). SRS emerges as the primary treatment option due to its favorable local control rates and improved neurocognitive outcomes compared to whole-brain radiotherapy (4).

As advancements in cancer treatment have led to increased patient survival, the significance of treatment-related side effects has become more pronounced. Radionecrosis stands out as a particularly critical side effect following brain SRS (5), and the concurrent use of SRS with newer-generation drugs remains an area with limited available information (6).

Trastuzumab emtansine is an antibody-drug conjugate (ADC) that combines trastuzumab (an anti-HER2 monoclonal antibody) with the cytotoxic agent DM1. Its ability to penetrate the central nervous system (CNS) is a topic of ongoing research and debate (7–9). While T-DM1 is not considered highly effective in penetrating an intact blood-brain barrier (BBB), it may reach brain metastases to some extent where the BBB is disrupted (8, 9). Therefore, while it can show some activity in the CNS under these conditions, its ability to penetrate the CNS is still relatively limited compared to other therapies like Tucatinib and lapatinib (1).

T-DM1 induces apoptosis by inhibiting microtubule polymerization and disrupting the cell cycle, which can explain previously reported cases of late hemorrhage and necrosis in the brain (10, 11).

In this report, we present the long-term toxicity and brain parenchymal changes observed in four cases of brain metastatic breast cancer treated with SRS and T-DM1. Our aim is to provide valuable insights into the potential late neurotoxicity associated with this treatment approach.

## Case 1

A 41-year-old patient was diagnosed with hormone-positive Her-2-positive breast cancer in 2009. After neoadjuvant chemotherapy, breast-conserving surgery and sentinel lymph node biopsy were done. In the pathology report, the complete response was achieved, and patients went to adjuvant breast radiation.

**Figure 1:**
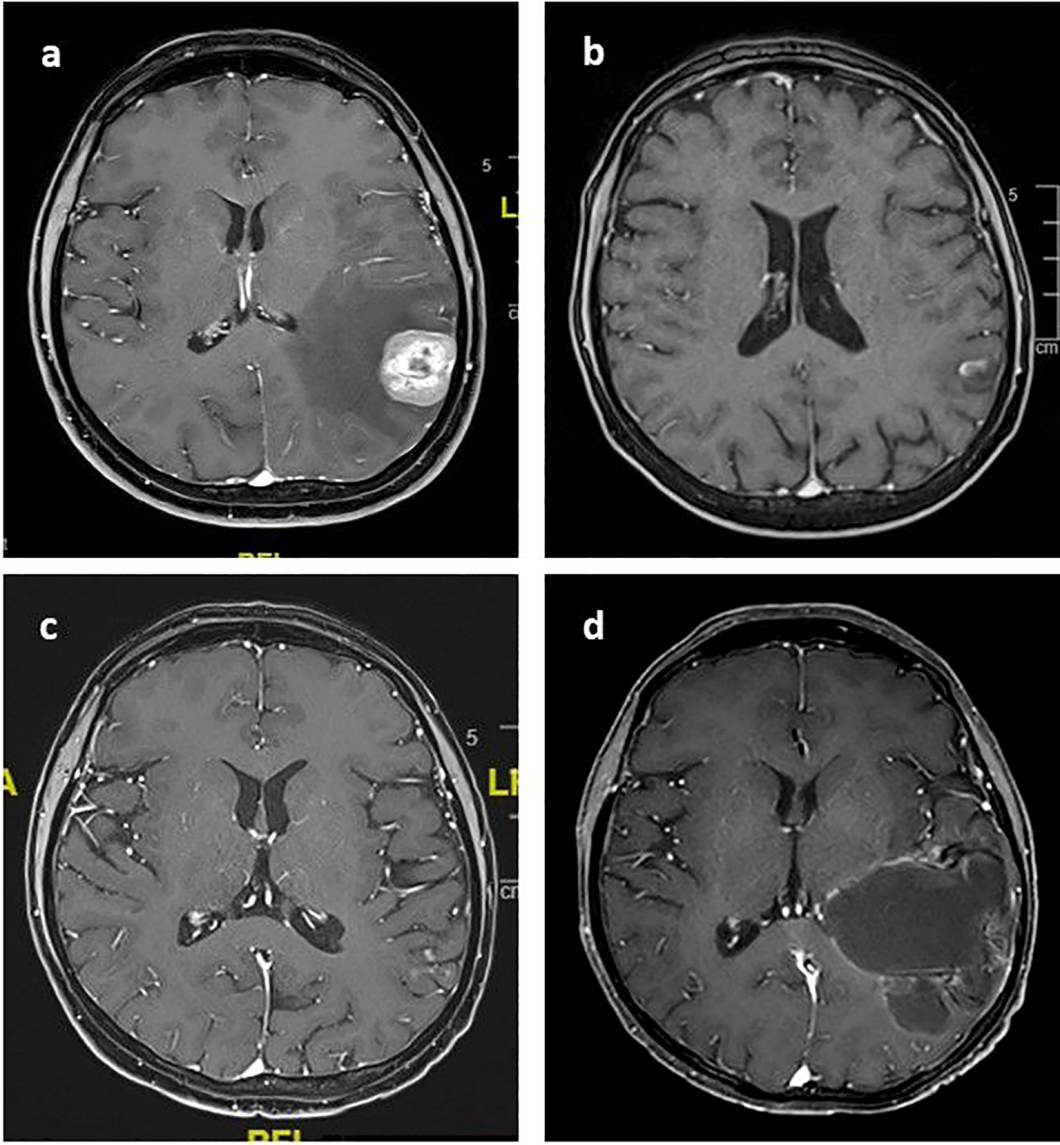
Post-contrast T1 axial images of the index lesion **(A)** Left temporal contrast-enhanced metastatic lesion treated with SRS in 2013 **(B)** near-total response six months after SRS **(C)** the maintenance of the local control when TDM1 was started in August 2020. **(D)** 58x62 mm cystic mass extends from the cortical surface to the ventricular level in January 2023.

The patient developed bone metastasis in 2011 and started chemotherapy along with palliative radiation to the bones. In 2013, she developed brain metastasis and underwent SRS with a single fraction dose of 18 Gy (**Figure 1A**). A near-complete response was achieved after six months (**Figure 1B**).

In October 2019, a brain MRI showed a new 5 mm metastasis in the left occipital region. Therefore, SRS was applied at 18 Gy in one fraction. The extracranial disease was under control with trastuzumab and pertuzumab.

In August 2020, the patient experienced progression in the right lung and started T-DM1. She was seen during regular follow-ups with controlled brain metastasis. The last brain MRI was in March 2021, after which she was lost to follow-up (**Figure 1C**).

The patient experienced neurological deterioration and difficulty speaking for the last two months as of January 2023. A brain MRI showed a partially contrast-enhanced parietotemporal cystic lesion on the left, with size progression, especially in the cystic components (**Figure 1D**). The patient was still receiving T-DM1 treatment during this period.

The patient was evaluated in a multidisciplinary tumor board, and surgery was recommended. Endoscopic cyst fenestration and biopsy were performed. The pathology report showed no viable tumor cells, only necrosis and necrobiosis.

## Case 2

A 29-year-old patient was diagnosed with hormone-negative, Her-2 positive bone-only metastatic breast cancer in 2015. After chemotherapy, a complete metabolic response was achieved in the bones. The patient underwent a subcutaneous mastectomy, sentinel lymph node biopsy, and radiation to the chest wall post-surgery.

In February 2018, the patient developed multiple brain metastases, and whole brain radiotherapy (WBRT) was applied. The systemic disease was controlled with trastuzumab.

**Figure 2:**
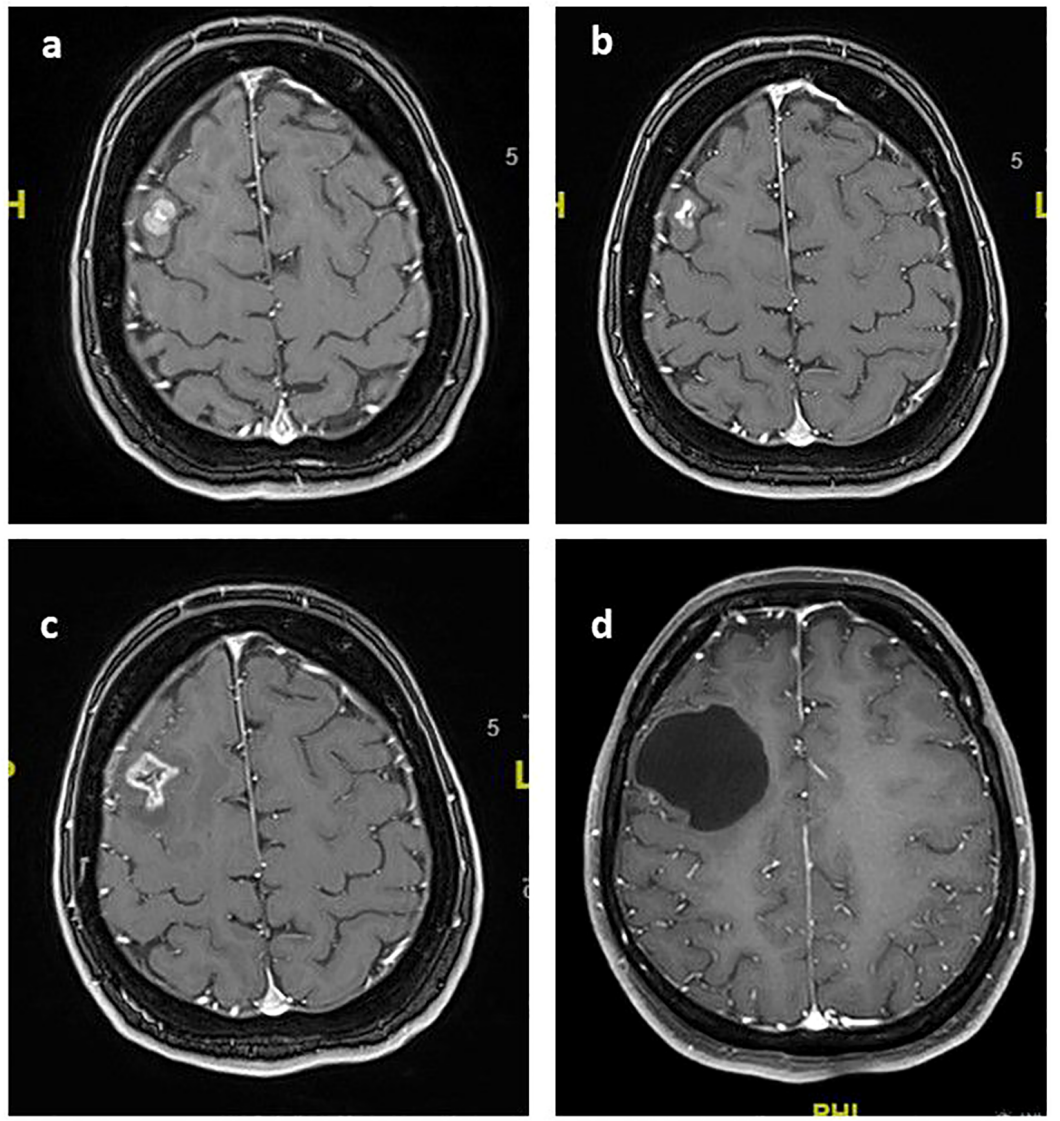
Post-contrast T1 axial images of the index lesion: **(A)** Right frontal contrast-enhanced metastatic lesion treated with SRS in February 2019 **(B)** near-total response when T-DM1 was started twelve months after SRS; **(C)** volumetric increase and peritumoral edema observed three months after starting T-DM1 in January 2020; **(D)** Stable 45x44 mm non-contrast-enhanced cystic mass in the right frontal lobe as of February 2023.

In February 2019, a new brain metastasis in the right frontal region was treated with SRS at a dose of 27 Gy in three fractions (**Figure 2A**). Additionally, in September 2019, two new lesions in the left parietal and right cerebellar regions were treated with single-fraction SRS at a dose of 18 Gy.

In January 2020, an ovarian metastasis was surgically resected, and T-DM1 treatment was initiated. The brain metastases remained under control (**Figure 2B**). After the initial treatment, the patient experienced worsening headaches and a volumetric increase in the size of previously treated lesions (**Figure 2C**). Perfusion imaging indicated these changes were due to brain necrosis. The patient had no neurological complaints except for mild headaches. Therefore, no medical treatment was administered following the detection of radionecrosis. During routine follow-up, the mild headache resolved, and no additional symptoms developed.The patient continued with regular follow-ups without any neurological symptoms. The last follow-up was in February 2023 (**Figure 2D**), showing that both intracranial and extracranial disease were under control.

## Case 3

**Figure 3:**
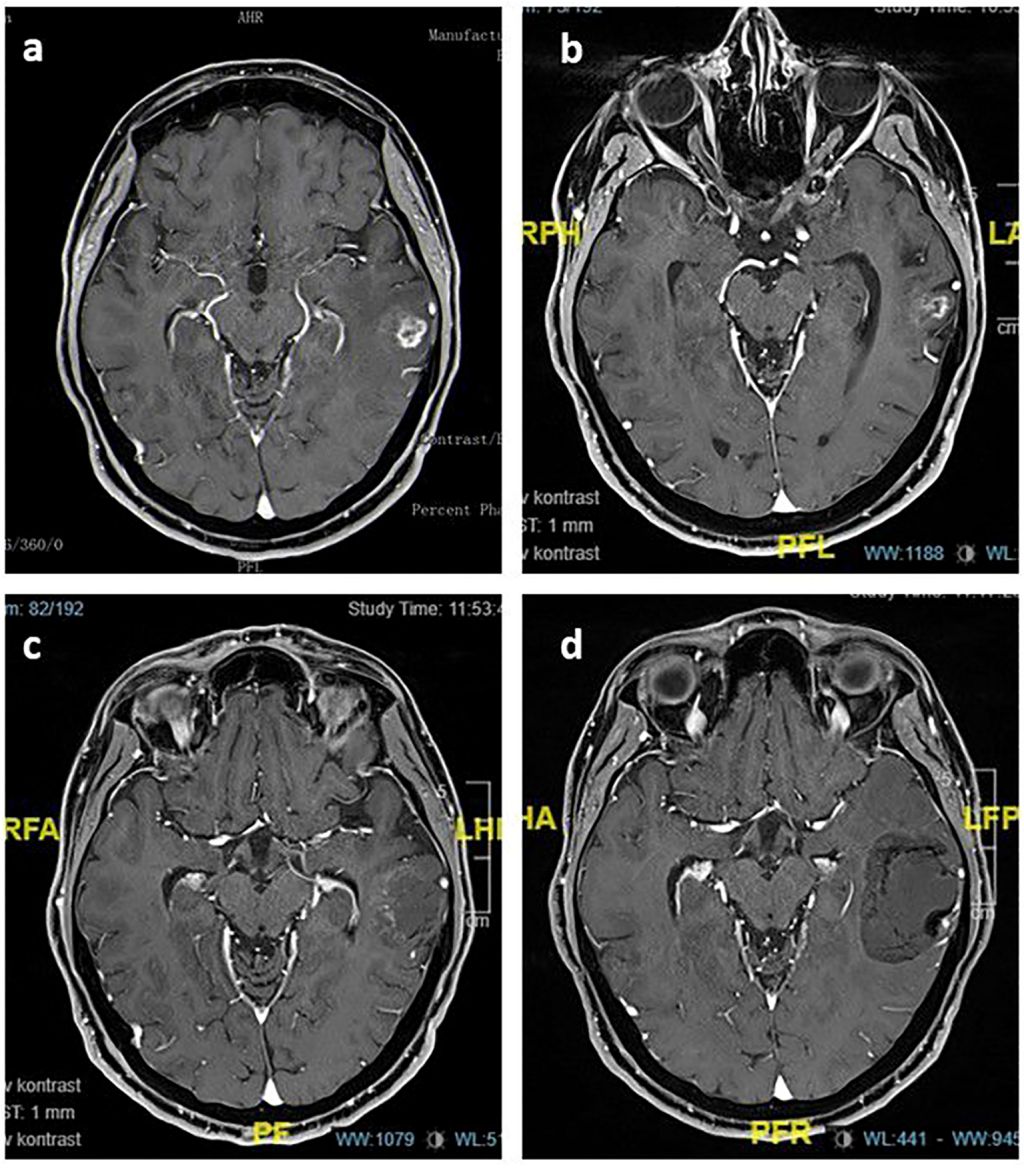
Post-contrast T1 axial images of the index lesion: **(A)** Left temporal contrast-enhanced metastatic lesion treated with SRS in May 2017; **(B)** controlled disease twelve months after SRS; **(C)** 28x34 mm mildly contrast-enhanced cystic mass in the left temporal lobe observed in December 2019; **(D)** 42x40 mm non-contrast-enhanced, stable cystic lesion in November 2022.

A 55-year-old patient was diagnosed with bone-only metastatic breast cancer, with a hormone-negative, Her-2 positive tumor subtype. Brain metastases developed in 2016 and were treated with SRS at a dose of 24 Gy in three fractions. In May 2017, a new left temporal metastatic lesion appeared (**Figure 3A**). The patient received 29 Gy in 3 fractions, and T-DM1 was started after brain SRS.

The patient was seen at regular follow-ups with controlled intracranial disease (**Figure 3B**). In December 2019, a cystic lesion appeared in the previously treated area without any mass effect or neurological symptoms (**Figure 3C**). Additionally, the patient underwent a perfusion MRI, which revealed hypoperfusion. Multiple brain lesions developed, and WBRT was applied in May 2020.

The last brain MRI in November 2022 showed controlled brain metastasis and a stable cystic lesion in the left temporal area (**Figure 3D**). At the most recent follow-up, the patient was still receiving T-DM1, having completed the 69th cycle, with extracranial disease under control.

## Case 4

A 40-year-old patient was diagnosed with hormone-negative, Her-2-positive breast cancer. After neoadjuvant treatment, the patient underwent surgery followed by irradiation of the left chest and lymphatic areas. Adjuvant trastuzumab was initiated for two years.

**Figure 4:**
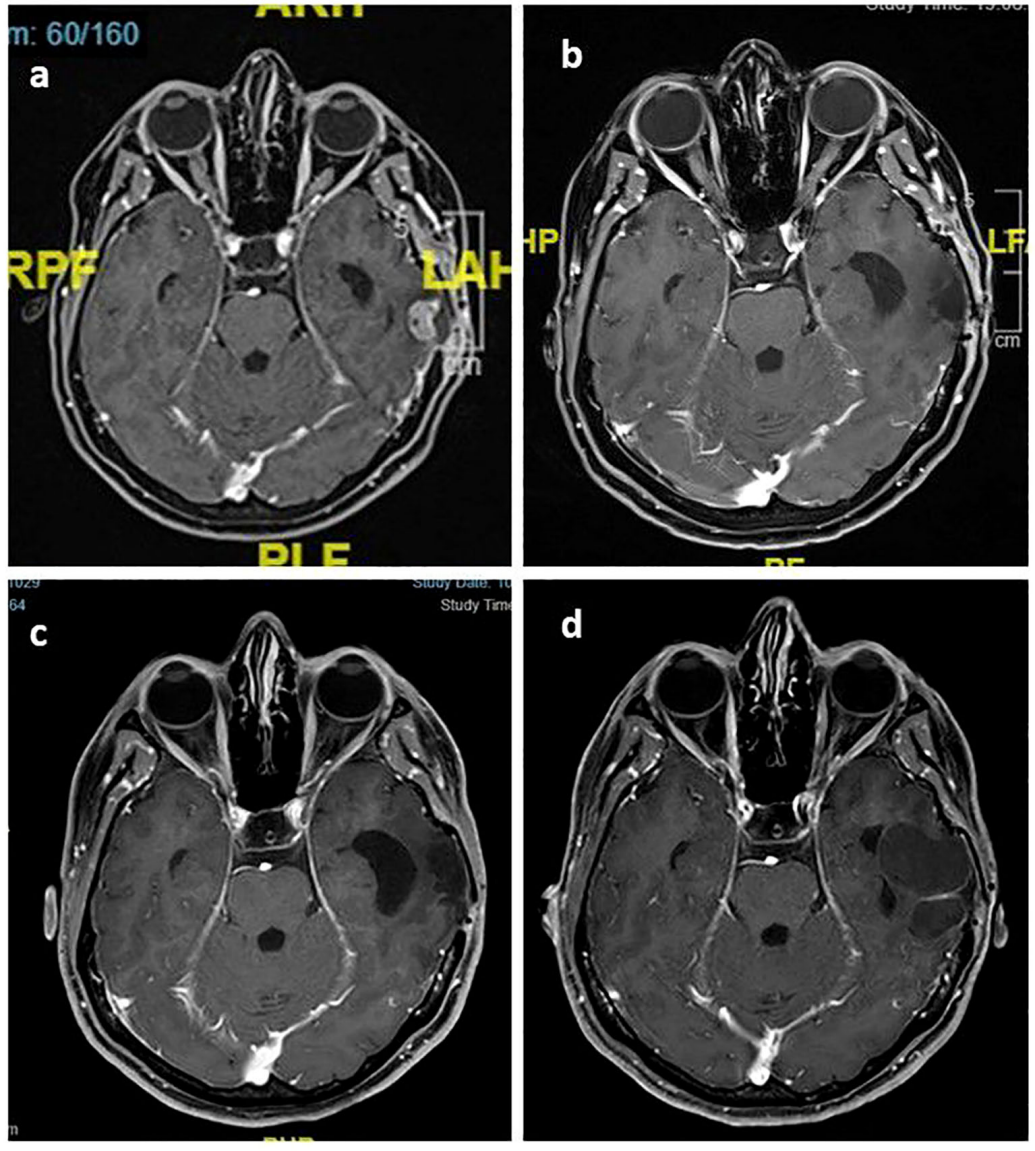
Post-contrast T1 axial images of the index lesion: **(A)** Left temporal contrast-enhanced metastatic lesion dated March 2019; **(B)** controlled disease six months after SRS; **(C)** maintenance of local control as of March 2022; **(D)** 42x40 mm non-contrast-enhanced, stable cystic lesion in March 2023.

In March 2019, the patient developed the first brain metastasis (**Figure 4A**). A left parietal mass was surgically resected, and the molecular profile was consistent with the initial diagnosis. SRS was applied in three fractions, totaling 27 Gy, to the residual lesion and the whole resection cavity (**Figures 4B, C**). The patient started systemic chemotherapy and trastuzumab, with no extracranial disease present.

In December 2019, new lesions appeared in the left temporal parenchyma and dural surfaces. SRS was applied to all lesions in five fractions, totaling 30 Gy, and T-DM1 was started. Since then, the patient has been seen at regular follow-ups without extracranial disease.

The last brain MRI in March 2023 showed a 33 x 15 mm cystic mass lesion with rim-style contrast enhancement (**Figure 4D**). Additionally, the patient underwent a perfusion MRI and no hyperperfusion was observed. Given the absence of neurological symptoms, no further treatment was recommended. During routine follow-up, the patient remained asymptomatic with the lesion stable as a cystic formation and still receiving T-DM1 treatment.

## Discussion

In this study, we presented four cases of brain metastatic breast cancer treated with SRS and T-DM1, highlighting the long-term toxicity and brain parenchymal changes observed. Our findings underscore the potential late neurotoxicity associated with this treatment approach, with a particular focus on the risk of radiation necrosis and its distinct radiological appearance.

Patients’ overall survival is prolonged with novel systemic therapies for different breast cancer subtypes and thus leading to an increase in the incidence of BMs (10). However, limited and complex information exists regarding the concurrent use of SRS with new-generation drugs (12). Currently, there is no specific guideline recommendation regarding the use of SRS for brain metastases in different breast cancer subtypes or with different targeted therapies. However, when there is a high risk of radiation necrosis, dose modification and fractionated SRS techniques can be considered based on general SRS literature (13). ASCO recommends prioritizing the combination of tucatinib, capecitabine, and trastuzumab for patients with HER2-positive metastatic breast cancer who have brain metastases, based on the HER2CLIMB and DESTINY trials (1, 14, 15). But ASTRO limits their recommendation to treating brain metastases with systemic drugs for a limited group, even if they have CNS transmission (4).

While increased toxicity has been reported when used concomitantly with certain drugs, this phenomenon has not been consistently observed across all anti-cancer medications (16–19). In the literature, T-DM1 has been reported to be associated with a high incidence of radiation necrosis following SRS (8, 11, 20). However, radiation necrosis associated with T-DM1 has not been reported with WBRT alone. In the study by Stumpf et al. involving a cohort of 45 patients with brain metastases from breast cancer, radiation necrosis developed in those who received T-DM1 either during or after SRS (21). This suggests a differential interaction between SRS and T-DM1. The same study indicated that T-DM1 enhanced the radiation-induced upregulation of aquaporin-4, a water transporter in astrocytes, leading to astrocytic swelling and an increase in astrocytic cell size at high radiation doses (21).

In our series of four cases, T-DM1 exhibited a distinct and characteristic radiological appearance pattern from a typical brain necrosis image characterized by necrotic foci, contrast enhancement, and perilesional edema (See in supplemantary). In all four cases, the lesions were consistently observed as enlarging cystic hemorrhagic masses on serial imaging. Furthermore, despite the presence of these post-treatment changes, there is no expected compressive mass effect associated with their size. SWI and T2 images exhibit peripheral signal drop-out, indicating the presence of hemosiderin deposition, which is commonly observed in hemorrhagic lesions associated with RT. This phenomenon has previously been reported by Mitsuya and colleagues in two patients exhibiting similar conditions. Following SRS for brain metastasis and subsequent administration of T-DM1 at the 13th and 14th month, progressive enlargement of the necrotic area was observed at the 5-year follow-up (11). Surgical resection revealed necrosis, hematoma, and granulation tissue upon pathological examination. The potential causes for these findings include neovascularization with associated microhemorrhages, T-DM1-induced telangiectasia, and thrombocytopenia.

Although the cumulative incidence of radiation necrosis (RN) shows an upward trend over time, the mean duration for RN development is typically around one year after SRS (3, 18). In our previous study involving a cohort of brain metastases from different primary sites, the median time to radiation necrosis was 12.7 months (ranging from 4.8 to 39.6) (3). In a retrospective study investigating the toxicity of brain SRS combined with T-DM1, an increased risk of radionecrosis was not observed during a median follow-up of 13 months, which aligns with the existing literature (19). Notably, in these four patients receiving T-DM1, radiation necrosis typically manifested in a delayed manner, occurring from the 45th month to the 10th year following SRS, and was triggered after the initiation of T-DM1 treatment. Furthermore, the data from our brain metastasis study revealed that no cases exhibited the typical large cystic radiation necrosis, which generally presents without mass effect and is predominantly either asymptomatic or only mildly symptomatic (3). Additionally in our multi-institutional retrospective study evaluating brain metastases from triple-negative breast cancer, we also did not observe an increased risk of radiation necrosis with the combination of current systemic therapies and brain SRS for any systemic medication (22). Based on this four-case series, we have designed a multi-institutional study to evaluate whether there is a rationale behind late and atypical radionecrosis after SRS in brain metastases among larger cohorts of HER2-positive breast cancer patients.

## Conclusion

T-DM1 may cause specific brain parenchymal changes and radiation necrosis in a late period in patients treated with brain SRS. Therefore, care should be taken regarding the risk of radionecrosis especially expected long-term survival and is crucial to assess the patient through a multidisciplinary approach.

## Data Availability

The raw data supporting the conclusions of this article will be made available by the authors, without undue reservation.
