# Supplementary figures and images for: Delayed neurotoxicity in HER2-positive breast cancer: a case series on combined SRS and T-DM1 treatment
Source: Front Oncol. 2024 Oct 1;14:1448593. doi: 10.3389/fonc.2024.1448593 (PMC11473412; doi:10.3389/fonc.2024.1448593)

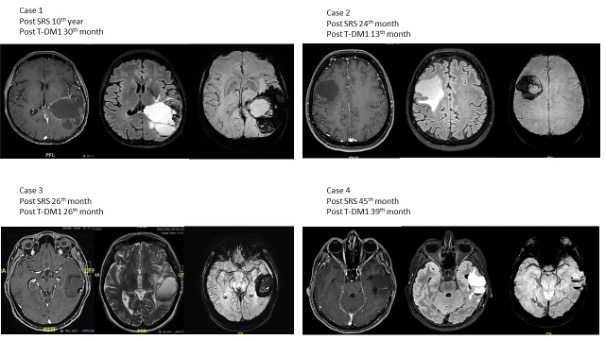

Supplement: Supplementary file 1 [file Image1.jpeg]
